# Integrative Genomic and Metabolomic Analysis Identifies mQTLs Associated with Genetic Selection for Tenderness in Nellore Cattle

**DOI:** 10.3390/metabo15120760

**Published:** 2025-11-25

**Authors:** Joao Marcos Bovetto de Campos Valim, Vinicius Laerte Silva Herreira, Ana Laura dos Santos Munhoz Gôngora, Lauro César Ferreira Beltrão, Eduardo Solano Pina dos Santos, Brenda Santos de Oliveira, Guilherme Pugliesi, Miguel Henrique de Almeida Santana, Guilherme Henrique Gebim Polizel, Luiz Alberto Colnago, Fernanda Maria Marins Ocampos, Germán Dário Ramírez-Zamudio, Saulo Luz Silva, Nara Regina Brandão Cônsolo

**Affiliations:** 1 Department of Animal Nutrition and Production, College of Veterinary Medicine and Animal Science, University of Sao Paulo, Pirassununga 13635-900, SP, Brazil; joaobovetto@usp.br (J.M.B.d.C.V.); vinicius.laerte@usp.br (V.L.S.H.); analauragongora@usp.br (A.L.d.S.M.G.); laurocesarbeltrao@usp.br (L.C.F.B.); brenda.soliv@usp.br (B.S.d.O.); fmmocampos@gmail.com (F.M.M.O.); 2 Department of Animal Science, School of Animal Science and Food Engineering, University of Sao Paulo, Pirassununga 13635-900, SP, Brazil; eduardosolano@usp.br (E.S.P.d.S.); mhasantana@usp.br (M.H.d.A.S.); guilherme.polizel@usp.br (G.H.G.P.); germanramvz@usp.br (G.D.R.-Z.); sauloluz@usp.br (S.L.S.); 3 Department of Animal Reproduction, College of Veterinary Medicine and Animal Science, University of Sao Paulo, Pirassununga 13635-900, SP, Brazil; gpugliesi@usp.br; 4 Brazilian Agricultural Research Corporation (EMBRAPA Instrumentation), São Carlos 13561-206, SP, Brazil; luiz.colnago@embrapa.br; 5 Center for Nutrition and Pregnancy, Department of Animal Sciences, North Dakota State University, Fargo, ND 58108, USA

**Keywords:** functional biomarkers, genotype–phenotype association, serum metabolites

## Abstract

Background: Beef tenderness is a key quality attribute that significantly influences consumer satisfaction; however, it exhibits considerable variability due to both genetic and environmental factors. While genomic selection based on Expected Progeny Differences (EPDs) has improved the accuracy of predictions, a substantial portion of tenderness variability remains unexplained. Metabolomics has emerged as a valuable approach to address this gap, as metabolites reflect gene–environment interactions and may serve as biomarkers for complex traits such as meat tenderness. Objectives: This study aimed to integrate genomic and metabolomic data to identify genetic loci associated with serum metabolites in Nellore calves, offspring of sires with contrasting EPDs for meat tenderness. Methods: Ninety-five male calves were evaluated and divided into two groups according to the sires’ genetic merit: FA-T (favorable EPD for tenderness, n = 45) and UN-T (unfavorable EPD for tenderness, n = 46). Blood serum samples were analyzed by ^1^H NMR spectroscopy to quantify 40 metabolites, and genotyping was performed using a medium-density SNP panel. Metabolite quantitative trait loci (mQTL) were identified using the MatrixEQTL package, and metabolic enrichment analysis was performed in MetaboAnalyst 6.0. Results: In the FA-T group, SNPs were associated with metabolites such as phenylalanine, tyrosine, and succinate, suggesting enhanced oxidative metabolism and preservation of proteolysis. In the UN-T group, associations of pyruvate, creatinine, and glutamine with distinct SNPs indicated greater reliance on anaerobic glycolysis and early ATP consumption, potentially impairing phosphorylation and postmortem proteolytic activity. Conclusions: These findings suggest that genetic selection for tenderness may induce early divergent metabolic profiles, likely leading to persistent differences in postmortem biochemical pathways, with important implications for meat tenderness.

## 1. Introduction

Beef tenderness is a complex and multifaceted trait influenced by both genetic and environmental factors, including management, nutrition, slaughter age, and pre-slaughter stress conditions [1,2]. Although only perceived during consumption, tenderness plays a key role in the consumer’s sensory experience and loyalty to the product [3,4]. However, within the beef industry, tenderness remains one of the major challenges associated with meat quality. Its high variability hinders industrial standardization and negatively impacts the commercial value of the product [5,6,7].

Since the 1990s, research on the genetic variability of beef tenderness has intensified, employing objective measurements such as Warner–Bratzler Shear Force (WBSF) and identifying the first genomic regions associated with tenderness in taurine breeds [8]. In the early 2000s, WBSF data began to be incorporated into genetic evaluations, leading to the development of Expected Progeny Difference (EPD) values specific to this trait [9,10]. Although tenderness exhibits moderate heritability, the accuracy of EPDs depends on extensive progeny records, which require considerable time and financial resources [11,12].

With the popularization of SNP panels in the late 2000s, genomic EPDs were developed, increasing the accuracy of genetic predictions in young animals, shortening the generation interval, and enhancing genetic gains [13,14,15,16]. In Brazil, however, genetic evaluations for meat tenderness remain limited due to the lack of large-scale carcass phenotypic data [17]. Nevertheless, breeding programs and academic studies have sought to associate tenderness with molecular markers and objective measures of meat quality [11,18,19,20,21,22,23].

Despite these advances, the variability associated with tenderness remains only partially explained by genetic markers, as environmental factors and genotype-by-environment interactions continue to represent sources of variation that are not fully captured [2]. In this context, metabolites, which are intermediate or end products of metabolism, directly reflect the interaction between the genome and the environment and may contribute to a better understanding and prediction of complex phenotypes [24,25], such as beef tenderness [26].

Metabolomics has been widely applied in animal production to evaluate metabolic responses to different nutritional strategies [27,28,29], to integrate with genetic improvement programs [24], and to investigate meat quality traits [30,31,32]. The integration of metabolomics with genomics, through the analysis of metabolic quantitative trait loci (mQTL), represents a promising approach for identifying functional biomarkers and supporting precision breeding strategies [33]. NMR-based metabolomics offers unique advantages including high reproducibility, direct quantification, minimal sample preparation, and the ability to capture comprehensive and non-destructive metabolic profiles, making it a robust and transparent tool for biomarker discovery. Although this approach has been extensively explored in human medicine to elucidate complex and multifactorial diseases [34,35], it remains scarcely applied in animal production, with reports limited to pigs [33,36] and no available studies in beef cattle focusing on meat quality traits.

In this context, this study hypothesizes that the selection of sires based on their EPDs for beef tenderness promotes genomic differences in their offspring, which are reflected in distinct serum metabolite profiles. Therefore, the objective of this study was to identify genomic loci associated with serum metabolites in Nellore calves, offspring of sires with contrasting estimated breeding values (EPDs) for meat tenderness, through mQTL analysis, aiming to integrate genomic and metabolomic data related to this trait.

## 2. Materials and Methods

Animal feeding management and data collection procedures were conducted at the School of Animal Science and Food Engineering of the University of São Paulo, located on the Fernando Costa Campus in Pirassununga, São Paulo, Brazil. The experimental protocol was approved by the Ethics Committee on Animal Use (CEUA) under approval number 9249180123.

### 2.1. Experimental Design and Calf Sampling

A total of 337 multiparous Nellore cows underwent fixed-time artificial insemination (FTAI) using semen from six Nellore bulls, selected based on their Expected Progeny Differences (EPD) for meat tenderness. The bulls represented the extremes of the genetic distribution for this trait within a 5% range. Three bulls exhibited superior genetic merit for tenderness (mean EPD: −0.0997; Accuracy: 0.69), while the remaining three showed inferior genetic merit (mean EPD: 0.0685; Accuracy: 0.61).

Male calves were assigned to two groups based on their sire’s expected progeny difference (EPD) for tenderness: FA-T (Favorable EPD for Meat Tenderness), comprising calves from bulls with favorable genetic values (n = 45), and UN-T (Unfavorable EPD for Meat Tenderness), comprising calves from bulls with unfavorable genetic values (n = 46) (Table 1).

Pregnant cows were maintained in pasture paddocks with *Urochloa brizantha* cv. Marandu, with free access to water, mineral supplements, and concentrate during the dry season. At birth, all calves from both groups received the same health and nutritional management. Blood samples and tail hair with follicles were collected from all calves at birth for metabolomic and genotyping analyses, respectively.

### 2.2. Processing of Blood Serum and Metabolite Extraction

Blood samples were collected in 10 mL tubes without anticoagulant (BD Vacutainer^®^, São Paulo, SP, Brazil), stored at 4 °C for 20 min and centrifuged at 2000× *g* for 15 min. The resulting serum was transferred to 2 mL Eppendorf tubes, properly labeled, and stored at −80 °C until macromolecule extraction was performed.

For sample preparation, 3 kDa filters (Amicon^®^ Ultra, Merck Millipore Ltd., Cork, Ireland) were used to remove macromolecular proteins and lipids, reducing spectral complexity, following the protocol described by Matias et al. [37]. Briefly, the filters were washed with 500 µL of Milli-Q water and centrifuged at 13,000× *g* for 5 min at 4 °C. This step was repeated five times, followed by a reverse spin at 7500× *g* for 60 s at 4 °C to remove residual water. Subsequently, 500 µL of serum were added to the filters and centrifuged at 13,000× *g* for 30 min at 4 °C to obtain the filtrate.

### 2.3. ^1^H NMR-Based Metabolomic Profiling

Filtrates were dried using a vacuum centrifuge and resuspended in 550 µL of phosphate buffer in D_2_O with 0.5 mM of DSS-d_6_ as an internal standard. The samples were then transferred to 5 mm nuclear magnetic resonance (NMR) tubes and analyzed using a Bruker Avance 14.1 T spectrometer equipped with a 5 mm Broadband Observe (BBO) probe (Bruker Corporation, Karlsruhe, Baden-Württemberg, Germany), observing ^1^H nuclei at 600.13 MHz.

One-dimensional ^1^H NMR spectra were acquired at 300K. A quality control sample, composed of a mixture of all serum samples, was used to calibrate the 90° pulse length, determine the offset for water suppression, and periodically monitor acquisition. Samples were randomly loaded into the NMR spectrometer, and ^1^H NMR spectra (128 transients and 64 K data points) were acquired using noesypr1d pulse sequence on a spectral width of ~20 ppm, with a mixing time of 100 ms and relaxation delay of 4 s. Spectra were processed using TopSpin 3.5 software. All ^1^H and ^13^C NMR chemical shifts were observed in ppm related to the DSS-d_6_ signal at 0.00 ppm as an internal reference, and an exponential line broadening of 0.3 Hz was applied. Following Fourier transformation, the spectra were automatically phased (zero and first phase), and the baseline was corrected.

Processing of the ^1^H-NMR spectra, as well as metabolite identification and quantification, was performed using 11.0 version Chenomx NMR Suite Professional software (Chenomx Inc., Edmonton, AB, Canada). During processing, the water signal region was removed, and phase, baseline, and spectral alignment corrections were applied using the DSS-d_6_ internal standard as a reference. Metabolite quantification was based on the ratio between the peak area of each metabolite and the DSS-d_6_ peak area, corresponding to a known concentration of 0.5 mM per sample.

### 2.4. Genotyping and SNP Quality Control

Genomic DNA previously extracted from the tail hair bulbs of the calves was used for genotyping with the Geneseek Genomic Profiler (GGP; NEOGEN Corporation, Lansing, MI, USA) *Bos indicus* 50K panel. Genotype calling was performed using GenomeStudio v2011.1, adopting a GenCall Score of 0.15 or greater, as recommended for assays based on Infinium chemistry, and applying the cluster file provided by the manufacturer. For each sample, overall genotyping quality was assessed by calculating the call rate, defined as the ratio between the number of called SNPs and the total number of SNPs in the panel.

SNPs were excluded if they had an unknown genomic position, were monomorphic, had a minor allele frequency (MAF) < 0.05, were located on sex chromosomes, exhibited a call rate < 95%, or deviated from Hardy–Weinberg equilibrium (*p* ≤ 10^−6^).

### 2.5. mQTL Identification and Enrichment Analysis

The identification of mQTL was performed using the MatrixEQTL package version 2.3 [38] in R software (version 4.4.0). Metabolomic data were previously normalized using Z-scores. MatrixEQTL evaluated associations between each SNP and each metabolite, considering genotypes as additive effects. Associations were tested individually, with false discovery rate correction (FDR) and a significance threshold of FDR < 0.05. No covariates were included in the model, as the study population consisted of a small and genetically homogeneous group of Nellore calves from a single herd, all managed under uniform environmental and nutritional conditions, minimizing the risk of spurious associations due to population stratification. Metabolic enrichment analysis was performed using the MetaboAnalyst 6.0 online platform, which applied the over-representation analysis function to the metabolites significantly associated with the groups exhibiting superior and inferior genetic merit for tenderness.

To identify potential candidate genes associated with significant SNPs from the mQTL analysis, we used GALLO v. 1.5 [39] within the R environment. This tool detects the genes located within a 500 kilobase window both upstream and downstream of the identified SNP regions. This strategy enabled a comprehensive mapping of genes in close proximity to the mQTLs, thereby providing a robust foundation for functional characterization.

## 3. Results

### 3.1. mQTL Profiles Associated with Favorable and Unfavorable EPD for Meat Tenderness

The genomic dataset initially comprised 51,976 SNPs. After filtering, two subsets were generated: 41,609 SNPs for the FA-T group and 41,614 SNPs for the UN-T group. ^1^H-NMR analysis produced a metabolomic dataset with 40 metabolites common to both groups.

**Figure 1:**
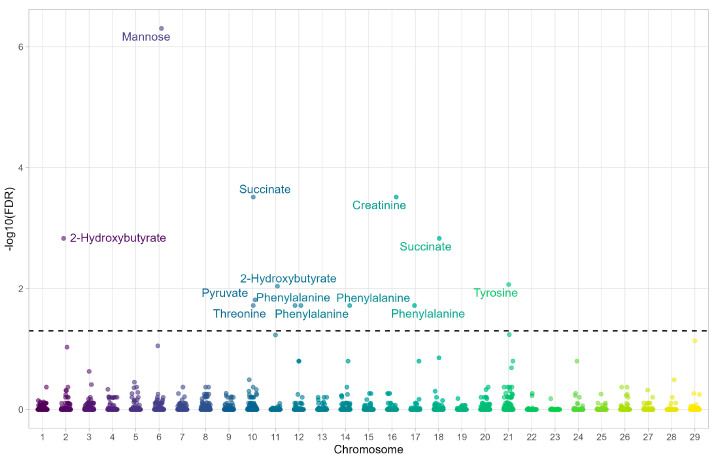
Manhattan plot showing the associations between metabolites and SNPs across chromosomes in the FA−T group (Favorable EPD for Meat Tenderness).

In the FA-T group, significant associations were identified between SNPs and metabolites (Figure 1, Table 2), including BovineHD0600010581–mannose (FDR = 4.971 × 10^−7^), BovineHD1000028745–succinate (FDR = 0.0003), BovineHD1600000258–creatinine (FDR = 0.0003), ARS-BFGL-NGS-109552–succinate (FDR = 0.001), ARS-BFGL-NGS-63882–2-hydroxybutyrate (FDR = 0.001), BovineHD2100016123–tyrosine (FDR = 0.008), ARS-BFGL-NGS-65835–2-hydroxybutyrate (FDR = 0.009), BovineHD1000028745–pyruvate (FDR = 0.015), ARS-BFGL-NGS-68102–threonine (FDR = 0.019), BovineHD1700013627–phenylalanine (FDR = 0.019), ARS-BFGL-NGS-32801–phenylalanine (FDR = 0.019), BovineHD1400003034–phenylalanine (FDR = 0.019), and Hapmap51939-BTA-21630–phenylalanine (FDR = 0.019).

**Figure 2:**
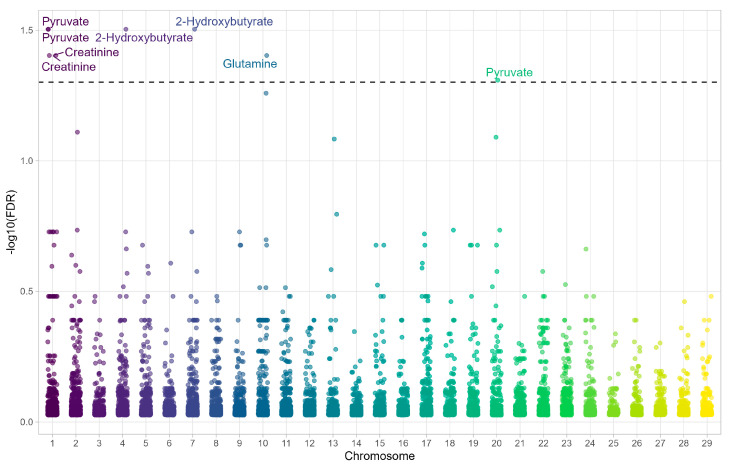
Manhattan plot showing the associations between metabolites and SNPs across chromosomes in the UF−T group (Unfavorable EPD for Meat Tenderness).

In the UN−T group (Figure 2, Table 3), noteworthy associations included: Bo-vineHD0100043594–pyruvate (FDR = 0.031), Hapmap57645-rs29027916–pyruvate (FDR = 0.031), BovineHD0400033104–2-hydroxybutyrate (FDR = 0.031), BovineHD0700014924–2-hydroxybutyrate (FDR = 0.031), Hapmap55240-rs29011308–glutamine (FDR = 0.039), BovineHD0100043594–creatinine (FDR = 0.039), Hapmap57645-rs29027916–creatinine (FDR = 0.039), and ARS-BFGL-NGS-107555–pyruvate (FDR = 0.049).

### 3.2. Pathway Enrichment Associated with Favorable and Unfavorable EPD for Meat Tenderness

**Figure 3:**
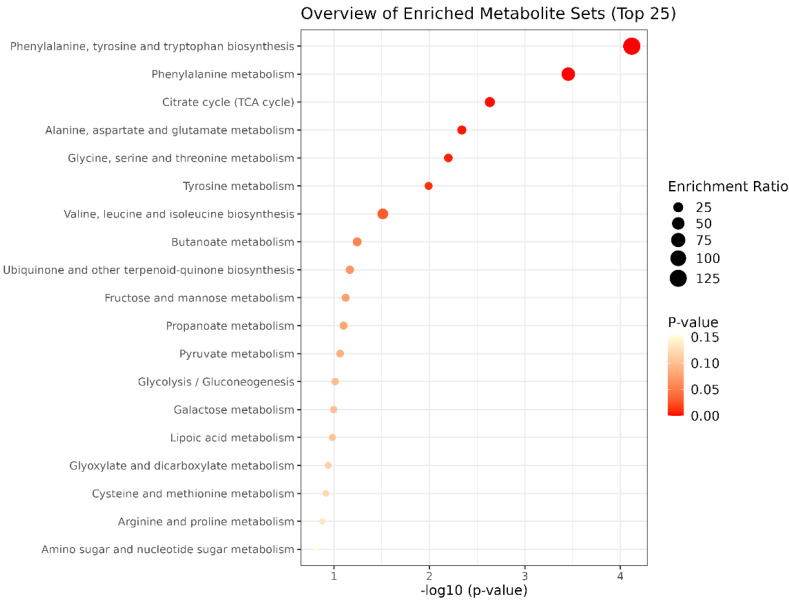
Pathway enrichment analysis of metabolites in the FA−T group (Favorable EPD for Tenderness).

In the FA−T group, the phenylalanine, tyrosine, and tryptophan biosynthesis pathway (FDR = 0.006) and the phenylalanine metabolism pathway (FDR = 0.014) were significantly enriched, as shown in Figure 3.

**Figure 4:**
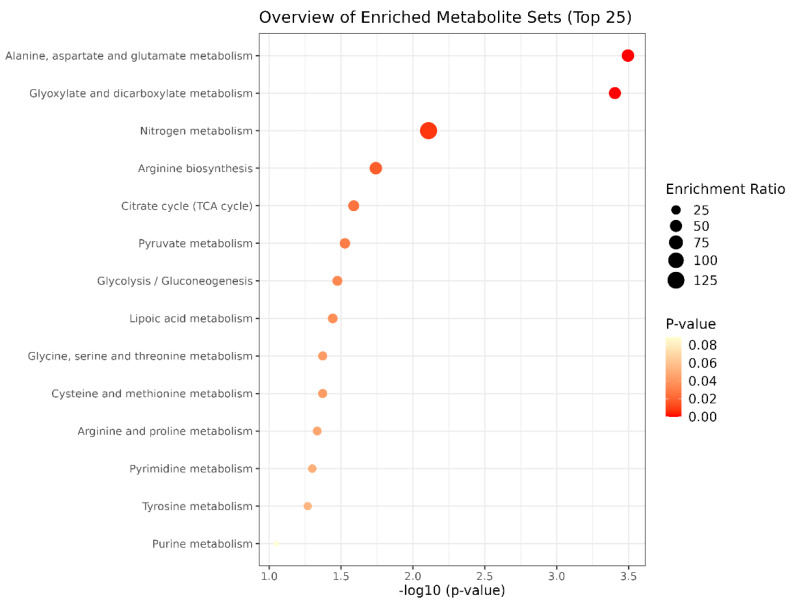
Pathway enrichment analysis of metabolites in the UF−T group (Unfavorable EPD for Meat Tenderness).

In the UN−T group, the alanine, aspartate, and glutamate metabolism pathway (FDR = 0.0157) and the glyoxylate and dicarboxylate metabolism pathway (FDR = 0.0157) stood out, as presented in Figure 4. The other metabolic pathways had FDR values > 0.05 and were not considered statistically significant.

**Figure 5:**
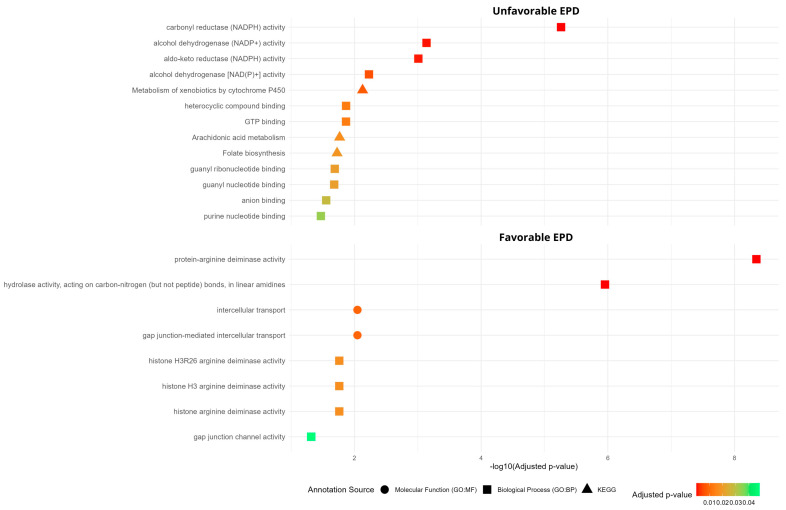
Pathway enrichment analysis of genes in the FA−T group (Favorable EPD for Tenderness) and UN−T group (Unfavorable EPD for Meat Tenderness).

Additionally, the genes dataset was used to identify enriched metabolic pathways (Figure 5). In the UN−T group, carbonyl reductase (NADPH) activity, alcohol dehydrogenase (NADP+) activity and aldo-keto reductase (NADPH) activity were the enriched biological processes (Figure 5). In contrast, in the FA−T group, protein-arginine deaminase activity and hydrolase activity, acting on carbon-nitrogen (but not peptide) bonds in linear amidines represented the enriched biological processes (Figure 5).

## 4. Discussion

In this study, we employed an integrated genomic and metabolomic approach to investigate the biological mechanisms underlying genetic selection for beef tenderness in Nelore cattle, using the progeny of sires with contrasting EPDs for this trait as the experimental model. The significant associations identified between SNPs and serum metabolite abundances provide evidence that specific metabolic profiles are influenced by particular genomic variants, revealing pathways potentially involved in the genetic selection for this trait.

To the best of our knowledge, this is the first study to integrate genomic and metabolomic data through mQTLs analysis in Nellore cattle, aiming to explore systemic metabolic mechanisms associated with genetic variability in the selection for meat quality traits. However, the data presented here are limited by the absence of direct assessment of the tenderness phenotype. Nevertheless, these results demonstrate how genetic selection for tenderness can influence muscle quantitative trait loci (mQTLs) and associated metabolic pathways. Consequently, to elucidate the functional relevance of the identified genes in meat tenderness, further functional validation is required in subsequent experiments, with comprehensive tenderness phenotyping prioritized as a central methodological criterion.

We observed that the progeny of genetically divergent sires (FA-T and UN-T groups) exhibited distinct mQTL profiles and enrichment of metabolic pathways, indicating that genetic selection for tenderness is reflected in systemic metabolism even at early developmental stages. In the FA-T group, SNPs from known genes [40] that showing significant associations with the metabolites were: ARS-BFGL-NGS-68102 (PRKCH), ARS-BFGL-NGS-32801 (RNF17), and Hapmap51939-BTA-21630 (PARP4). For the UN-T group, the SNPs were: Hapmap57645-rs29027916 (CHAF1B), BovineHD0100043594 (MORC3), BovineHD0400033104 (GBX1), and BovineHD0700014924 (CDC25C). None of these specific genes have been previously reported in relation to the genetic selection of the phenotype of meat tenderness [11]. However, chromosomes 1, 4, 6, 7, 8, 10, 18, 19, 20, 21, 22, 25, 26 and 29 have been identified as regions influencing meat tenderness in Nellore cattle. The associated SNPs in the FA-T group are located on chromosomes 2, 6, 10, 11, 12, 14, 16, 17, 18 and 21, while those in the UN-T group are on chromosomes 1, 4, 7, 10 and 20, which aligns with some of the chromosomes previously linked to this trait.

The identification of specific associations between SNPs and metabolites—such as mannose, phenylalanine, succinate, threonine, and tyrosine in the FA-T group, and glutamine in the UN-T group—supports the hypothesis that genotype-mediated regulatory mechanisms distinctly modulate metabolic pathways involved in genetic selection for meat tenderness. Although the present data do not directly reflect the tenderness phenotype, the possibility that genetic selection modulates the expression of genes and mQTLs associated with this trait remains. If these genetic and molecular changes persist throughout the animal’s life, they may induce alterations in the muscle’s biochemical state prior to the onset of rigor mortis, thereby influencing postmortem metabolic pathways and ultimately affecting meat quality via shifts in metabolite profiles and energy metabolism. Nevertheless, it remains unknown whether the observed gene, mQTL, or metabolite changes identified in calves persist into adulthood.

Nevertheless, some of the metabolites described in this study have previously been associated with the tenderness phenotype in other contexts. In metabolomic studies conducted directly on beef muscle, phenylalanine, threonine, and tyrosine were found in higher abundance in samples classified as tender, showing a positive correlation with desmin degradation and a negative correlation with shear force [41]. In the FA-T group, threonine was associated with ARS-BFGL-NGS-68102. This SNP is linked to the PRKCH gene, which encodes proteins of the protein kinase C (PKC) family. These proteins play crucial roles in cellular processes such as protection against hypoxia, cell regulation, signaling, and intercellular adhesion [42]. The activation of PKC proteins requires threonine [43], which further substantiates the association between this metabolite and the SNP. Given the correlation of threonine with muscle degradation [41], this observation can be linked to the cellular adhesion function of the proteins synthesized by the PRKCH gene and a potential beneficial effect on meat tenderness in this effect is maintained throughout the late stages of the animal’s life.

Phenylalanine was associated with two distinct SNPs in the FA-T group: ARS-BFGL-NGS-32801, which is linked to the RNF17 gene, and Hapmap51939-BTA-21630, associated with PARP4. The RNF17 gene encodes the ring finger 17 protein in germ cells, which is essential for animal reproduction [44] and has no known association with genetic selection or the tenderness phenotype. In the UN-T group, serum glutamine concentration was associated with a SNP; its abundance in beef has previously been positively correlated with tenderness [31]. However, in postmortem muscle tissue, glutamine synthesis occurs through the conversion of glutamate in an ATP-dependent process [45]. This additional energy consumption may reduce ATP availability for other biochemically relevant processes in muscle metabolism, potentially influencing the phosphorylation rate of myofibrillar proteins and, indirectly, affecting the activity of proteolytic systems such as calpain, with possible negative repercussions for meat tenderness [46]. However, based on these data, it cannot be guaranteed that these metabolic differences will persist throughout the later stages of the animals’ lives, potentially resulting in variation in the tenderness phenotype.

FA-T calves showed a specific association between the SNP BovineHD0600010581 and mannose. D-mannose, the C-2 epimer of D-glucose, has been linked to increased glycogen degradation during the postmortem period in beef quality studies [47,48] and also demonstrates a positive relationship with pyruvate concentrations [48]. In this study, both FA-T and UN-T calves exhibited mQTLs associated with pyruvate, albeit involving distinct SNPs, suggesting that different genetic regulatory mechanisms may converge on the same metabolic pathway. Notably, the UN-T group exhibited associations with three SNPs associated with pyruvate (BovineHD0100043594, Hapmap57645-rs29027916, and ARS-BFGL-NGS-107555), while the FA-T group presented only one (BovineHD1000028745). Pyruvate is a central metabolite in glucose metabolism, participating in glycolysis, gluconeogenesis, and the tricarboxylic acid (TCA) cycle [49]. After slaughter, its role in postmortem muscle acidification is critical for the onset rigor and meat properties [50], the greater diversity of mQTLs in the UN-T group does not necessarily indicate greater glycolytic efficiency. Conversely, this pattern may reflect genomic regulation distributed across multiple regions, suggesting a potentially more complex and less efficient control over this metabolite [51].

Similarly, two SNPs related to creatinine (BovineHD0100043594 and Hapmap57645-rs29027916) were associated with UN-T group, compared to only one SNP (BovineHD1600000258) associated with FA-T group. Creatinine, a product of phosphocreatine degradation, is considered an indirect marker of energy mobilization during periods of high muscle metabolic demand [52,53]. Although this evidence is based on evaluations in live animals, it may have important implications in the postmortem context. In this scenario, the creatine/phosphocreatine system serves as the primary pathway for immediate ATP generation in skeletal muscle, acting as an acid–base buffer by consuming H^+^ ions [54,55]. However, this system is rapidly depleted, and the subsequent decrease in ATP levels, accompanied by ADP accumulation, activates glycolysis as the main remaining energy pathway [56,57]. This process results in lactate production and muscle acidification, both of which are essential for the development of meat texture [55]. Creatine has previously been assossiated with beef tenderenss in studies evaluating the tenderness phenotype [31]. In the present study, serum creatinine levels measured in newborn calves may indirectly reflect the activity of the phosphagen system throughout the animals’ development, which, in the long term, could influence the rate of postmortem pH decline and the activation ofproteolytic enzymes. However, the greater diversity of creatinine-related mQTLs in the UN-T group may indicate more complex genomic regulation of this energy system, though not necessarily greater efficiency, with potential repercussions for muscle acidification dynamics and, consequently, meat tenderness.

In the present study, three SNPs were identified with simultaneous associations to two distinct metabolites, suggesting shared genetic regulation or co-association, as they displayed highly significant FDR values for both metabolites. In the FA-T group, the SNP BovineHD1000028745 was significantly associated with pyruvate (FDR = 0.015), and the SNP BovineHD1000028745 and ARS-BFGL-NGS-109552 with succinate (FDR = 0.0003 0.001, respectively), both central intermediates of energy metabolism involved in glycolysis pathway and the TCA cycle, respectively [58]. In postmortem muscle, pyruvate is converted to lactate under anaerobic conditions; however, in the presence of residual oxygen, it can be oxidized by mitochondria for ATP production [58]. Additionally, succinate, a TCA cycle intermediate, is abundant in the mitochondrial matrix and is widely used in oxidative metabolism postmortem [58,59]. In a study evaluating the tenderness phenotype, the authors suggested that increased mitochondrial activity may precede oxidative stress, rendering structural proteins more susceptible to myofibrillar proteolysis, which favors meat tenderness [31].

On the other hand, in the UN-T group, the SNPs BovineHD0100043594 and Hapmap57645-rs29027916 were simultaneously associated with pyruvate (FDR = 0.031 and 0.031, respectively) and creatinine (FDR = 0.039 and 0.039, respectively). The *CHAF1B* gene is functionally involved in nucleosome assembly and organization, while *MORC3* is implicated in cellular senescence and proliferation, as well as protein localization and stability [60]. Although no association have been reported for these genes in studies of genettic selection or meat quality phenotypes. Pyruvate and creatine are key metabolites in muscle energy metabolism. Creatinine, a degradation product of creatine phosphate, serve as a marker of phosphagen system activation [52,53]. During the conversion of muscle to meat, this system, together with glycolysis, provides a temporary source of ATP [61]. However, both processes gradually fail to maintain energy homeostasis in postmortem tissue, leading to ATP depletion [61]. In contrast oxidative metabolism generates reactive oxygen species that promote the opening of the mitochondrial permeability transition pore [62]. This effect is exacerbated by imbalances between ATP production and consumption, increasing cytosolic calcium levels and causing mitochondrial calcium overload [62,63]. Consequently, following *rigor mortis*, inhibition of calcium uptake by the mitochondrial matrix accelerates calpain activation, thereby promoting meat tenderization [64].

Compared with the association of pyruvate and succinate with the same SNP observed in FA-T calves, the simultaneous association of pyruvate and creatinine with two common SNPs in UN-T group suggests a more glycolysis-oriented metabolic profile. Although pyruvate and creatinine are present in both groups of calves in this study, differences in genetic associations, particularly the exclusive presence of succinate in the FA-T group, indicate that these metabolites are part of distinct postmortem metabolic pathways. While multivariate or correlation analyses between allelic effects were not conducted, these findings provide initial evidence of candidate loci that warrant functional investigation and further validation in future studies.

In the present study, 2-hydroxybutyrate was associated with two distinct SNPs in the FA-T group (ARS-BFGL-NGS-63882; ARS-BFGL-NGS-65835) and with two SNPs in the UN-T group (BovineHD0400033104; BovineHD0700014924), suggesting that its genomic regulation occurs via different mechanisms between the evaluated groups. No gene association were identified for the SNPs linked to the FA-T group. However, those from the UF-T group were associated with the GBX1 gene (BovineHD0400033104) and CDC25C (BovineHD0700014924). The GBX1 gene is involved in protease inhibition and modulates stress response pathways, thereby enhancing cellular resistance [65]. 2-hydroxybutyrate is a metabolite derived from the degradation of methionine and threonine, and is a by-product of the glutathione synthesis pathway, generated when cystathionine is converted into cysteine [66]. Although there is still no direct evidence linking 2-hydroxybutyrate to genetic selection of meat quality traits such as tenderness, its role as a by-product of glutathione synthesis allows for physiologically grounded hypotheses. In skeletal muscle, enhanced antioxidant capacity may improve cell membrane stability, thereby reducing glycolysis rate [67] and, consequently, postmortem acidification [68].

Considering that the UN-T calves in this study exhibited a serum metabolic profile indicative of higher glycolytic activity, as previously discussed, the concurrent presence of 2-hydroxybutyrate may reflect a compensatory antioxidant response to metabolic stress. In this context, when extrapolated to postmortem muscle metabolism, this response could limit the progression of glycolysis, thereby influencing the rate of pH decline and, consequently, meat tenderness. In contrast, in FA-T calves, whose profile is associated with more oxidative metabolism, the serum abundance of 2-hydroxybutyrate, an indicator of antioxidant activity, may be synergistic with the prevailing biochemical environment and may not compromise the processes responsible for meat tenderization.

However, it is important to note tha the primary limitation of this study is the absence of direct measurements of beef tenderness; therefore, no causal relationship can yet be established between the identified genes, mQTLs, or metabolites and the tenderness phenotype. Additionally, the relatively small sample size (FA-T n = 45; UN-T n = 46; total n = 91) may have limited the statistical power of the mQTL analysis, increasing the likelihood of false negatives despite the application of multiple-testing correction to control false positives. Future studies with larger and independent populations, incorporating direct measures of meat tenderness, are essential to validate and expand upon the associations reported here. Despite these limitations, this study provides a novel integrative approach combining genomic and metabolomic data in Nellore cattle, offering valuable insights into the early biological mechanisms that may underlie differences associated with genetic selection for beef tenderness.

## 5. Conclusions

This study identified mQTLs and metabolic pathways associated with groups differing genetic potential for meat tenderness in Nellore cattle, suggesting that genetic selection for this trait may induce distinct metabolic profiles at an early stage. The FA-T group appears to exhibit greater energetic support and enhanced postmortem proteolysis, whereas the UN-T group shows higher glycolytic dependence and a greater risk of energetic limitation, potentially affecting protein degradation. As previously mentioned, these results are not supported by the tenderness assessments. However, if such physiological differences persist throughout the final stages of the animal’s life, they may lead to modifications in the muscle’s biochemical state prior to the onset of rigor mortis. This may, in turn, influence postmortem metabolic pathways and ultimately affect meat quality through changes in metabolite profiles and energy metabolism. These findings highlight the potential of genetically modulated metabolites as candidate biomarkers, although their predictive value requires validation in future studies that incorporate direct tenderness phenotyping.

## Figures and Tables

**Table 1:** Number of progenies from each sire and treatments.

Sire	Progeny, n	Genetic Selection for Tenderness		
		Treatment	EPD	Accuracy
Sire 1	15	FA-T	−0.1217	0.79
Sire 2	13	FA-T	−0.0974	0.63
Sire 3	17	FA-T	−0.08	0.66
Total	45	Mean	−0.0997	0.69
Sire 4	24	UF-T	0.0751	0.65
Sire 5	13	UF-T	0.0773	0.6
Sire 6	9	UF-T	0.0531	0.59
Total	46	Mean	0.0685	0.61

**Table 2:** Known genes associations between SNPs and metabolites in the FA−T group (Favorable EPD to Meat Tenderness).

SNP	Metabolite	Gene	Chromosome	FDR
ARS-BFGL-NGS-68102	Threonine	*PRKCH*	10	0.019
ARS-BFGL-NGS-32801	Phenylalanine	*RNF17*	12	0.019
Hapmap51939-BTA-21630	Phenylalanine	*PARP4*	12	0.019

**Table 3:** Known genes associations between SNPs and metabolites in the UF−T group (Unfavorable EPD to Meat Tenderness).

SNP	Metabolite	Gene	Chromosome	FDR
Hapmap57645-rs29027916	Pyruvate	*CHAF1B*	1	0.031
BovineHD010 0043594	Pyruvate	*MORC3*	1	0.031
BovineHD040 0033104	2-hydroxybutyrate	*GBX1*	4	0.031
BovineHD070 0014924	2-hydroxybutyrate	*CDC25C*	7	0.031
BovineHD010 0043594	Creatinine	*MORC3*	1	0.039
Hapmap57645-rs29027916–	Creatinine	*CHAF1B*	1	0.039

## Data Availability

Data will be available under request.

## Abbreviations

The following abbreviations are used in this manuscript:

ATP	Adenosine Triphosphate
CEUA	Ethics Committee on Animal Use
DSS-d_6_	4,4-Dimethyl-4-silapentane-1-sulfonic acid-d_6_ (internal standard for NMR)
EPD	Expected Progeny Difference
FA-T	Favorable EPD for Tenderness
FDR	False Discovery Rate
FTAI	Fixed-Time Artificial Insemination
MAF	Minor Allele Frequency
mQTL	Metabolic Quantitative Trait Locus
NMR	Nuclear Magnetic Resonance
ppm	Parts per million
SNP	Single Nucleotide Polymorphism
UF-T	Unfavorable EPD for Tenderness
WBSF	Warner–Bratzler Shear Force
^1^H NMR	Proton Nuclear Magnetic Resonance
TXI	Triple Resonance Inverse detection probe

## References

[1] Robinson D.L., Ferguson D.M., Oddy V.H., Perry D., Thompson J. (2001). Genetic and Environmental Influences on Beef Tenderness. Aust. J. Exp. Agric..

[2] Warner R.D., Greenwood P.L., Pethick D.W., Ferguson D.M. (2010). Genetic and Environmental Effects on Meat Quality. Meat Sci..

[3] Drey L.N., Legako J.F., Brooks J.C., Miller M.F., O’quinn T.G. (2017). The Contribution of Tenderness, Juiciness, and Flavor to Overall Consumer Beef Eating Experience. Meat Muscle Biol..

[4] Warner R.D., Wheeler T.L., Ha M., Li X., Bekhit A.E.-D., Morton J., Vaskoska R., Dunshea F.R., Liu R., Purslow P. (2022). Meat Tenderness: Advances in Biology, Biochemistry, Molecular Mechanisms and New Technologies. Meat Sci..

[5] Boleman S.L., Boleman S.J., Morgan W.W., Hale D.S., Griffin D.B., Savell J.W., Ames R.P., Smith M.T., Tatum J.D., Field T.G. (1998). National Beef Quality Audit-1995: Survey of Producer-Related Defects and Carcass Quality and Quantity Attributes. J. Anim. Sci..

[6] McKenna D.R., Roebert D.L., Bates P.K., Schmidt T.B., Hale D.S., Griffin D.B., Savell J.W., Brooks J.C., Morgan J.B., Montgomery T.H. (2002). National Beef Quality Audit-2000: Survey of Targeted Cattle and Carcass Characteristics Related to Quality, Quantity, and Value of Fed Steers and Heifers. J. Anim. Sci..

[7] O’Quinn T.G., Legako J.F., Brooks J.C., Miller M.F. (2018). Evaluation of the Contribution of Tenderness, Juiciness, and Flavor to the Overall Consumer Beef Eating Experience1. Transl. Anim. Sci..

[8] Koohmaraie M., Wheeler T.L., Shackelford S.D. (1995). Beef Tenderness: Regulation and Prediction.

[9] Garrick D. (2005). Trends and Developments in Genetic Evaluation of Beef Cattle in the United States. Proceedings of the 9th World Angus Forum Technical Meeting: Angus in the Global Market.

[10] Bolsen J.W., Bormann J.M., Moser D.W., Marston T.T. (2006). Relationship between Sire Tenderness EPD and Progeny Carcass Performance. J. Anim. Sci..

[11] Carvalho M.E., Baldi F.S., Santana M.H.A., Ventura R.V., Oliveira G.A., Bueno R.S., Bonin M.N., Rezende F.M., Coutinho L.L., Eler J.P. (2017). Identification of Genomic Regions Related to Tenderness in Nellore Beef Cattle. Adv. Anim. Biosci..

[12] Regatieri I.C., Boligon A.A., Baldi F., Albuquerque L.G. (2012). Genetic Correlations between Mature Cow Weight and Productive and Reproductive Traits in Nellore Cattle. Genet. Mol. Res..

[13] Van Eenennaam A.L., Li J., Thallman R.M., Quaas R.L., Dikeman M.E., Gill C.A., Franke D.E., Thomas M.G. (2007). Validation of Commercial DNA Tests for Quantitative Beef Quality Traits1,2. J. Anim. Sci..

[14] Wiggans G.R., Cole J.B., Hubbard S.M., Sonstegard T.S. (2017). Genomic Selection in Dairy Cattle: The USDA Experience. Annu. Rev. Anim. Biosci..

[15] Hayes B.J., Lewin H.A., Goddard M.E. (2013). The Future of Livestock Breeding: Genomic Selection for Efficiency, Reduced Emissions Intensity, and Adaptation. Trends Genet..

[16] Smith K.V., DeLong K.L., Griffith A.P., Boyer C.N., Martinez C., Jensen K.L. (2023). Cow-Calf Producer Preferences for Bull Genomic Enhanced Expected Progeny Differences. J. Agric. Resour. Econ..

[17] Castro L.M.d., Magnabosco C.U., Sainz R.D., Faria C.U.d., Lopes F.B. (2014). Quantitative Genetic Analysis for Meat Tenderness Trait in Polled Nellore Cattle. Rev. Ciência Agronômica.

[18] Braz C.U., Taylor J.F., Bresolin T., Espigolan R., Feitosa F.L.B., Carvalheiro R., Baldi F., de Albuquerque L.G., de Oliveira H.N. (2019). Sliding Window Haplotype Approaches Overcome Single SNP Analysis Limitations in Identifying Genes for Meat Tenderness in Nelore Cattle. BMC Genet..

[19] Carvalho M.E., Eler J.P., Bonin M.N., Rezende F.M., Biase F.H., Meirelles F.V., Regitano L.C.A., Coutinho L.L., Balieiro J.C.C., Ferraz J.B.S. (2017). Genotypic and Allelic Frequencies of Gene Polymorphisms Associated with Meat Tenderness in Nellore Beef Cattle. Genet. Mol. Res..

[20] Gonçalves T.M., de Almeida Regitano L.C., Koltes J.E., Cesar A.S.M., da Silva Andrade S.C., Mourão G.B., Gasparin G., Moreira G.C.M., Fritz-Waters E., Reecy J.M. (2018). Gene Co-Expression Analysis Indicates Potential Pathways and Regulators of Beef Tenderness in Nellore Cattle. Front. Genet..

[21] McClure M.C., Ramey H.R., Rolf M.M., McKay S.D., Decker J.E., Chapple R.H., Kim J.W., Taxis T.M., Weaber R.L., Schnabel R.D. (2012). Genome-wide Association Analysis for Quantitative Trait Loci Influencing Warner–Bratzler Shear Force in Five Taurine Cattle Breeds. Anim. Genet..

[22] Pinto L.F.B., Ferraz J.B.S., Pedrosa V.B., Eler J.P., Meirelles F.V., Bonin M.N., Rezende F.M., Carvalho M.E., Cucco D.C., Silva R.C.G. (2011). Single Nucleotide Polymorphisms in CAPN and Leptin Genes Associated with Meat Color and Tenderness in Nellore Cattle. Genet. Mol. Res..

[23] Pinto L.F.B., Ferraz J.B.S., Meirelles F.V., Eler J.P., Rezende F.M., Carvalho M.E., Almeida H.B., Silva R.C.G. (2010). Association of SNPs on CAPN1 and CAST Genes with Tenderness in Nellore Cattle. Genet. Mol. Res..

[24] Fontanesi L. (2016). Metabolomics and Livestock Genomics: Insights into a Phenotyping Frontier and Its Applications in Animal Breeding. Anim. Front..

[25] Goldansaz S.A., Guo A.C., Sajed T., Steele M.A., Plastow G.S., Wishart D.S. (2017). Livestock Metabolomics and the Livestock Metabolome: A Systematic Review. PLoS ONE.

[26] D’Alessandro A., Marrocco C., Rinalducci S., Mirasole C., Failla S., Zolla L. (2012). Chianina Beef Tenderness Investigated through Integrated Omics. J. Proteom..

[27] Magan J.B., O’Callaghan T.F., Zheng J., Zhang L., Mandal R., Hennessy D., Fenelon M.A., Wishart D.S., Kelly A.L., McCarthy N.A. (2019). Impact of Bovine Diet on Metabolomic Profile of Skim Milk and Whey Protein Ingredients. Metabolites.

[28] Ramírez-Zamudio G.D., Silva L.H.P., Vieira N.M., Vilela R.S.R., Assis D.E.F., Assis G.J.F., Estrada M.M., Rodrigues R.T.S., Duarte M.S., Chizzotti M.L. (2022). Effect of Short-Term Dietary Protein Restriction before Slaughter on Meat Quality and Skeletal Muscle Metabolomic Profile in Culled Ewes. Livest. Sci..

[29] Polizel G.H.G., Cançado F.A.C.Q., Dias E.F.F., Fernandes A.C., Cracco R.C., Carmona B.T., Castellar H.H., Poleti M.D., Santana M.H.d.A. (2022). Effects of Different Prenatal Nutrition Strategies on the Liver Metabolome of Bulls and Its Correlation with Body and Liver Weight. Metabolites.

[30] Antonelo D.S., Cônsolo N.R.B., Gómez J.F.M., Beline M., Goulart R.S., Corte R.R.P.S., Colnago L.A., Schilling M.W., Gerrard D.E., Silva S.L. (2020). Metabolite Profile and Consumer Sensory Acceptability of Meat from Lean Nellore and Angus × Nellore Crossbreed Cattle Fed Soybean Oil. Food Res. Int..

[31] Antonelo D., Gómez J.F.M., Cônsolo N.R.B., Beline M., Colnago L.A., Schilling W., Zhang X., Suman S.P., Gerrard D.E., Balieiro J.C.C. (2020). Metabolites and Metabolic Pathways Correlated With Beef Tenderness. Meat Muscle Biol..

[32] Cônsolo N.R.B., Rosa A.F., Barbosa L.C.G.S., Maclean P.H., Higuera-Padilla A., Colnago L.A., Titto E.A.L. (2021). Preliminary Study on the Characterization of Longissimus Lumborum Dark Cutting Meat in Angus × Nellore Crossbreed Cattle Using NMR-Based Metabolomics. Meat Sci..

[33] Bovo S., Ribani A., Fanelli F., Galimberti G., Martelli P.L., Trevisi P., Bertolini F., Bolner M., Casadio R., Dall’Olio S. (2025). Merging Metabolomics and Genomics Provides a Catalog of Genetic Factors That Influence Molecular Phenotypes in Pigs Linking Relevant Metabolic Pathways. Genet. Sel. Evol..

[34] Dumas M.-E., Wilder S.P., Bihoreau M.-T., Barton R.H., Fearnside J.F., Argoud K., D’Amato L., Wallis R.H., Blancher C., Keun H.C. (2007). Direct Quantitative Trait Locus Mapping of Mammalian Metabolic Phenotypes in Diabetic and Normoglycemic Rat Models. Nat. Genet..

[35] Nicholson G., Rantalainen M., Li J.V., Maher A.D., Malmodin D., Ahmadi K.R., Faber J.H., Barrett A., Min J.L., Rayner N.W. (2011). A Genome-Wide Metabolic QTL Analysis in Europeans Implicates Two Loci Shaped by Recent Positive Selection. PLoS Genet..

[36] Ponsuksili S., Trakooljul N., Hadlich F., Methling K., Lalk M., Murani E., Wimmers K. (2019). Genetic Regulation of Liver Metabolites and Transcripts Linking to Biochemical-Clinical Parameters. Front. Genet..

[37] Matias I.F.B., Santos E.S.P., Valim J.M.B.d.C., Castro A., Ferreira A.G., Barbosa L.C., Ribeiro G.H., Colnago L.A., Asnchau D.G., Souza Y.G. (2025). da S.; et al. Preparation of Ruminal Fluid and Serum Samples from Beef Cattle for Nuclear Magnetic Resonance Based–Metabolomics. N. Z. J. Agric. Res..

[38] Shabalin A.A. (2012). Matrix EQTL: Ultra Fast EQTL Analysis via Large Matrix Operations. Bioinformatics.

[39] Fonseca P.A.S., Suárez-Vega A., Marras G., Cánovas Á. (2020). GALLO: An R Package for Genomic Annotation and Integration of Multiple Data Sources in Livestock for Positional Candidate Loci. Gigascience.

[40] Zimin A.V., Delcher A.L., Florea L., Kelley D.R., Schatz M.C., Puiu D., Hanrahan F., Pertea G., Van Tassell C.P., Sonstegard T.S. (2009). A Whole-Genome Assembly of the Domestic Cow, Bos Taurus. Genome Biol..

[41] King D.A., Shackelford S.D., Broeckling C.D., Prenni J.E., Belk K.E., Wheeler T.L. (2019). Metabolomic Investigation of Tenderness and Aging Response in Beef Longissimus Steaks. Meat Muscle Biol..

[42] Garcia-Concejo A., Larhammar D. (2021). Protein Kinase C Family Evolution in Jawed Vertebrates. Dev. Biol..

[43] Seki T., Matsubayashi H., Amano T., Shirai Y., Saito N., Sakai N. (2005). Phosphorylation of PKC Activation Loop Plays an Important Role in Receptor-mediated Translocation of PKC. Genes Cells.

[44] Fernández J.C., Pérez J.E., Herrera N., Martínez R., Bejarano D., Rocha J.F. (2019). Research Article Genomic Association Study for Age at First Calving and Calving Interval in Romosinuano and Costeño Con Cuernos Cattle. Genet. Mol. Res..

[45] Ouali A., Gagaoua M., Boudida Y., Becila S., Boudjellal A., Herrera-Mendez C.H., Sentandreu M.A. (2013). Biomarkers of Meat Tenderness: Present Knowledge and Perspectives in Regards to Our Current Understanding of the Mechanisms Involved. Meat Sci..

[46] Ren C., Hou C., Zhang D., Li X., Xiao X., Bai Y. (2021). ATP Regulates the Phosphorylation and Degradation of Myofibrillar Proteins in Ground Ovine Muscle. J. Integr. Agric..

[47] Koutsidis G., Elmore J.S., Oruna-Concha M.J., Campo M.M., Wood J.D., Mottram D.S. (2008). Water-Soluble Precursors of Beef Flavour: I. Effect of Diet and Breed. Meat Sci..

[48] Giovanini de Oliveira Sartori A., Silva Antonelo D., Ribeiro G.H., Colnago L.A., de Carvalho Balieiro J.C., Francisquine Delgado E., Contreras Castillo C.J. (2024). Lipidome and Metabolome Profiling of Longissimus Lumborum Beef with Different Ultimate PH and Postmortem Aging. Meat Sci..

[49] Lu G., Li Y., Mao K., Zang Y., Zhao X., Qiu Q., Qu M., Ouyang K. (2022). Effects of Rumen-Protected Creatine Pyruvate on Meat Quality, Hepatic Gluconeogenesis, and Muscle Energy Metabolism of Long-Distance Transported Beef Cattle. Front. Anim. Sci..

[50] Lomiwes D., Farouk M.M., Wu G., Young O.A. (2014). The Development of Meat Tenderness is Likely to Be Compartmentalised by Ultimate PH. Meat Sci..

[51] Chan E.K.F., Rowe H.C., Hansen B.G., Kliebenstein D.J. (2010). The Complex Genetic Architecture of the Metabolome. PLoS Genet..

[52] Liao Y., Hu R., Wang Z., Peng Q., Dong X., Zhang X., Zou H., Pu Q., Xue B., Wang L. (2018). Metabolomics Profiling of Serum and Urine in Three Beef Cattle Breeds Revealed Different Levels of Tolerance to Heat Stress. J. Agric. Food Chem..

[53] Serrano-Contreras J.I., García-Pérez I., Meléndez-Camargo M.E., Zepeda L.G. (2016). NMR-Based Metabonomic Analysis of Physiological Responses to Starvation and Refeeding in the Rat. J. Proteome Res..

[54] Li J.L., Guo Z.Y., Li Y.J., Zhang L., Gao F., Zhou G.H. (2016). Effect of Creatine Monohydrate Supplementation on Carcass Traits, Meat Quality and Postmortem Energy Metabolism of Finishing Pigs. Anim. Prod. Sci..

[55] Wicks J., Beline M., Gomez J.F.M., Luzardo S., Silva S.L., Gerrard D. (2019). Muscle Energy Metabolism, Growth, and Meat Quality in Beef Cattle. Agriculture.

[56] Bendall J.R. (1951). The Shortening of Rabbit Muscles during Rigor Mortis: Its Relation to the Breakdown of Adenosine Triphosphate and Creatine Phosphate and to Muscular Contraction. J. Physiol..

[57] Bate-Smith E.C., Bendall J.R. (1949). Factors Determining the Time Course of Rigor Mortis. J. Physiol..

[58] England E.M., Matarneh S.K., Mitacek R.M., Abraham A., Ramanathan R., Wicks J.C., Shi H., Scheffler T.L., Oliver E.M., Helm E.T. (2018). Presence of Oxygen and Mitochondria in Skeletal Muscle Early Postmortem. Meat Sci..

[59] Matarneh S.K., Yen C.-N., Bodmer J., El-Kadi S.W., Gerrard D.E. (2021). Mitochondria Influence Glycolytic and Tricarboxylic Acid Cycle Metabolism under Postmortem Simulating Conditions. Meat Sci..

[60] Rocha R.d.F.B., Garcia A.O., Otto P.I., dos Santos M.G., da Silva M.V.B., Martins M.F., Machado M.A., Panetto J.C.d.C., Guimarães S.E.F. (2023). Single-Step Genome-Wide Association Studies and Post-GWAS Analyses for the Number of Oocytes and Embryos in Gir Cattle. Mamm. Genome.

[61] Scheffler T.L., Gerrard D.E. (2007). Mechanisms Controlling Pork Quality Development: The Biochemistry Controlling Postmortem Energy Metabolism. Meat Sci..

[62] Haworth R.A., Hunter D.R. (1979). The Ca^2+^-Induced Membrane Transition in Mitochondria. Arch. Biochem. Biophys..

[63] Ramos P.M., Li C., Elzo M.A., Wohlgemuth S.E., Scheffler T.L. (2020). Mitochondrial Oxygen Consumption in Early *Postmortem* Permeabilized Skeletal Muscle Fibers Is Influenced by Cattle Breed. J. Anim. Sci..

[64] Dang D.S., Buhler J.F., Davis H.T., Thornton K.J., Scheffler T.L., Matarneh S.K. (2020). Inhibition of Mitochondrial Calcium Uniporter Enhances Postmortem Proteolysis and Tenderness in Beef Cattle. Meat Sci..

[65] Buckley D.M., Burroughs-Garcia J., Kriks S., Lewandoski M., Waters S.T. (2020). Gbx1 and Gbx2 Are Essential for Normal Patterning and Development of Interneurons and Motor Neurons in the Embryonic Spinal Cord. J. Dev. Biol..

[66] Tsoukalas D., Fragoulakis V., Papakonstantinou E., Antonaki M., Vozikis A., Tsatsakis A., Buga A.M., Mitroi M., Calina D. (2020). Prediction of Autoimmune Diseases by Targeted Metabolomic Assay of Urinary Organic Acids. Metabolites.

[67] den Hertog-Meischke M.J.A., Smulders F.J.M., Houben J.H., Eikelenboom G. (1997). The Effect of Dietary Vitamin E Supplementation on Drip Loss of Bovine Longissimus Lumborum, Psoas Major and Semitendinosus Muscles. Meat Sci..

[68] Dou L., Jin Y., Li H., Liu C., Yang Z., Chen X., Sun L., Zhao L., Su L. (2023). Effect of Feeding System on Muscle Fiber Composition, Antioxidant Capacity, and Nutritional and Organoleptic Traits of Goat Meat. Animals.

